# Association of Genetic Variants Affecting microRNAs and Pancreatic Cancer Risk

**DOI:** 10.3389/fgene.2021.693933

**Published:** 2021-08-30

**Authors:** Ye Lu, Chiara Corradi, Manuel Gentiluomo, Evangelina López de Maturana, George E. Theodoropoulos, Susanne Roth, Evaristo Maiello, Luca Morelli, Livia Archibugi, Jakob R. Izbicki, Patricia Sarlós, Vytautas Kiudelis, Martin Oliverius, Mateus Nóbrega Aoki, Yogesh Vashist, Casper H. J. van Eijck, Maria Gazouli, Renata Talar-Wojnarowska, Andrea Mambrini, Raffaele Pezzilli, Bas Bueno-de-Mesquita, Péter Hegyi, Pavel Souček, John P. Neoptolemos, Gregorio Di Franco, Cosimo Sperti, Emanuele F. Kauffmann, Viktor Hlaváč, Faik G. Uzunoğlu, Stefano Ermini, Ewa Małecka-Panas, Maurizio Lucchesi, Giuseppe Vanella, Frederike Dijk, Beatrice Mohelníková-Duchoňová, Franco Bambi, Maria Chiara Petrone, Krzysztof Jamroziak, Feng Guo, Katerina Kolarova, Giovanni Capretti, Anna Caterina Milanetto, Laura Ginocchi, Martin Loveček, Marta Puzzono, Hanneke W. M. van Laarhoven, Silvia Carrara, Audrius Ivanauskas, Konstantinos Papiris, Daniela Basso, Paolo G. Arcidiacono, Ferenc Izbéki, Roger Chammas, Pavel Vodicka, Thilo Hackert, Claudio Pasquali, Maria L. Piredda, Eithne Costello-Goldring, Giulia Martina Cavestro, Andrea Szentesi, Francesca Tavano, Barbara Włodarczyk, Hermann Brenner, Edita Kreivenaite, Xin Gao, Stefania Bunduc, Roel C. H. Vermeulen, Martin A. Schneider, Anna Latiano, Domenica Gioffreda, Sabrina G. G. Testoni, Juozas Kupcinskas, Rita T. Lawlor, Gabriele Capurso, Núria Malats, Daniele Campa, Federico Canzian

**Affiliations:** ^1^Genomic Epidemiology Group, German Cancer Research Center (DKFZ), Heidelberg, Germany; ^2^Medical Faculty Heidelberg, University of Heidelberg, Heidelberg, Germany; ^3^Department of Biology, University of Pisa, Pisa, Italy; ^4^Genetic and Molecular Epidemiology Group, Spanish National Cancer Research Centre (CNIO), Madrid, Spain; ^5^First Propaedeutic University Surgery Clinic, Hippocratio General Hospital, Medical School, National and Kapodistrian University of Athens, Athens, Greece; ^6^Department of General Surgery, University of Heidelberg, Heidelberg, Germany; ^7^Department of Oncology, Fondazione IRCCS “Casa Sollievo della Sofferenza” Hospital, San Giovanni Rotondo, Italy; ^8^General Surgery, Department of Translational Research and New Technologies in Medicine and Surgery, University of Pisa, Pisa, Italy; ^9^Digestive and Liver Disease Unit, Sant’Andrea Hospital, Rome, Italy; ^10^Faculty of Medicine and Psychology, Sapienza University of Rome, Rome, Italy; ^11^Pancreato-Biliary Endoscopy and Endosonography Division, Pancreas Translational and Clinical Research Center, IRSSC San Raffaele Scientific Institute, Milan, Italy; ^12^Department of General, Visceral and Thoracic Surgery, University Medical Center Hamburg-Eppendorf, Hamburg, Germany; ^13^First Department of Medicine, Medical School, University of Pécs, Pécs, Hungary; ^14^Department of Gastroenterology, Institute for Digestive Research, Medical Academy, Lithuanian University of Health Sciences, Kaunas, Lithuania; ^15^Department of Surgery, Faculty Hospital Kralovske Vinohrady and Third Faculty of Medicine, Charles University, Prague, Czechia; ^16^Laboratory for Applied Science and Technology in Health, Carlos Chagas Institute, Curitiba, Brazil; ^17^Department of Surgery, Erasmus Medical Center, Erasmus University, Rotterdam, Netherlands; ^18^Laboratory of Biology, Medical School, National and Kapodistrian University of Athens, Athens, Greece; ^19^Department of Digestive Tract Diseases, Medical University of Lodz, Lodz, Poland; ^20^Oncological Department, Azienda USL Toscana Nord Ovest, Oncological Unit of Massa Carrara, Carrara, Italy; ^21^Department of Gastroenterology, San Carlo Hospital, Potenza, Italy; ^22^Department for Determinants of Chronic Diseases (DCD), National Institute for Public Health and the Environment (RIVM), Bilthoven, Netherlands; ^23^Institute for Translational Medicine, Medical School, University of Pécs, Pécs, Hungary; ^24^Department of Medicine, Centre for Translational Medicine, University of Szeged, Szeged, Hungary; ^25^Biomedical Center, Faculty of Medicine in Pilsen, Charles University, Pilsen, Czechia; ^26^Department of Surgery-DiSCOG, Padua University Hospital, Padua, Italy; ^27^Division of General and Transplant Surgery, University of Pisa, Pisa, Italy; ^28^Blood Transfusion Service, Azienda Ospedaliero-Universitaria Meyer, Children's Hospital, Florence, Italy; ^29^Deparment of Pathology, Cancer Center Amsterdam, Amsterdam University Medical Centers, University of Amsterdam, Amsterdam, Netherlands; ^30^Department of Oncology, Faculty of Medicine and Dentistry, Palacky University Olomouc and University Hospital Olomouc, Olomouc, Czechia; ^31^Department of Hematology, Institute of Hematology and Transfusion Medicine, Warsaw, Poland; ^32^Division of Clinical Epidemiology and Aging Research, German Cancer Research Center (DKFZ), Heidelberg, Germany; ^33^Department of Biomedical Sciences, Humanitas University, Milan, Italy; ^34^Pancreatic Surgery Unit, Humanitas Clinical and Research Center IRCCS, Milan, Italy; ^35^Department of Surgery I, Faculty of Medicine and Dentistry, Palacky University Olomouc and University Hospital Olomouc, Olomouc, Czechia; ^36^Division of Experimental Oncology, Gastroenterology and Gastrointestinal Endoscopy Unit, Vita-Salute San Raffaele University, IRCCS San Raffaele Scientific Institute, Milan, Italy; ^37^Department of Medical Oncology, Cancer Center Amsterdam, Amsterdam University Medical Center, University of Amsterdam, Amsterdam, Netherlands; ^38^Division of Gastroenterology and Digestive Endoscopy, Department of Gastroenterology, Humanitas Clinical and Research Center IRCCS, Milan, Italy; ^39^Endoscopic Surgery Department, Hippocratio General Hospital of Athens, Athens, Greece; ^40^Department of Medicine-DIMED, Padua University Hospital, Padua, Italy; ^41^Szent György University Teaching Hospital of County Fejér, Székesfehérvár, Hungary; ^42^Department of Radiology and Oncology, Institute of Cancer of São Paulo (ICESP), São Paulo, Brazil; ^43^Faculty of Medicine, University of São Paulo, São Paulo, Brazil; ^44^Department of Molecular Biology of Cancer, Institute of Experimental Medicine of the Czech Academy of Sciences, Prague, Czechia; ^45^Biomedical Centre and Department of Surgery, Faculty of Medicine in Pilsen, Charles University, Pilsen, Czechia; ^46^First Faculty of Medicine, Institute of Biology and Medical Genetics, Charles University, Prague, Czechia; ^47^ARC-NET, Centre for Applied Research on Cancer, University and Hospital Trust of Verona, Verona, Italy; ^48^Department of Molecular and Clinical Cancer Medicine, University of Liverpool, Liverpool, United Kingdom; ^49^Division of Gastroenterology and Research Laboratory, Fondazione IRCCS “Casa Sollievo della Sofferenza” Hospital, San Giovanni Rotondo, Italy; ^50^Division of Preventive Oncology, German Cancer Research Center (DKFZ) and National Center for Tumor Diseases (NCT), Heidelberg, Germany; ^51^German Cancer Consortium (DKTK), German Cancer Research Center (DKFZ), Heidelberg, Germany; ^52^Fundeni Clinical Institute, Bucharest, Romania; ^53^Institute for Risk Assessment Sciences (IRAS), Utrecht University, Utrecht, Netherlands

**Keywords:** pancreatic cancer, miRNA, genetic polymorphisms, susceptibility, pancreatic ductal adenocarcinoma

## Abstract

Genetic factors play an important role in the susceptibility to pancreatic cancer (PC). However, established loci explain a small proportion of genetic heritability for PC; therefore, more progress is needed to find the missing ones. We aimed at identifying single nucleotide polymorphisms (SNPs) affecting PC risk through effects on micro-RNA (miRNA) function. We searched *in silico* the genome for SNPs in miRNA seed sequences or 3 prime untranslated regions (3'UTRs) of miRNA target genes. Genome-wide association data of PC cases and controls from the Pancreatic Cancer Cohort (PanScan) Consortium and the Pancreatic Cancer Case–Control (PanC4) Consortium were re-analyzed for discovery, and genotyping data from two additional consortia (PanGenEU and PANDoRA) were used for replication, for a total of 14,062 cases and 11,261 controls. None of the SNPs reached genome-wide significance in the meta-analysis, but for three of them the associations were in the same direction in all the study populations and showed lower value of *p* in the meta-analyses than in the discovery phase. Specifically, rs7985480 was consistently associated with PC risk (OR = 1.12, 95% CI 1.07–1.17, *p* = 3.03 × 10^−6^ in the meta-analysis). This SNP is in linkage disequilibrium (LD) with rs2274048, which modulates binding of various miRNAs to the 3'UTR of *UCHL3*, a gene involved in PC progression. In conclusion, our results expand the knowledge of the genetic PC risk through miRNA-related SNPs and show the usefulness of functional prioritization to identify genetic polymorphisms associated with PC risk.

## Background

Pancreatic cancer (PC) incidence is rising, and it is still one of the most fatal cancers due to the absence of early symptoms, the lack of early detection methods, and limited effect of surgical resection even when in the context of multimodal treatments. Several epidemiologic PC risk factors have been identified, including cigarette smoking, heavy alcohol intake, type two diabetes mellitus, high BMI, and chronic pancreatitis (Maisonneuve and Lowenfels, 2015; Barone et al., 2016; Principe and Rana, 2020). The genetic susceptibility to PC is explained by rare high penetrance mutations identified through sequencing approaches (Hu et al., 2018) and common low penetrance variants discovered through genome-wide association studies (GWAS; Amundadottir et al., 2009; Low et al., 2010; Petersen et al., 2010; Wu et al., 2012; Wolpin et al., 2014; Childs et al., 2015; Zhang et al., 2016; Klein et al., 2018; Campa et al., 2020; Gentiluomo et al., 2020; Lin et al., 2020; López de Maturana et al., 2021) or candidate gene approaches (Campa et al., 2013a, 2015, 2016b; Feng et al., 2019; Gentiluomo et al., 2019a,b; Xu et al., 2019; Yang et al., 2019; Corradi et al., 2021). Only a small number of susceptibility loci for PC risk have been found thus far. Besides, previously established risk loci explain only a small proportion of genetic heritability for PC, and it is estimated that there may be over 1,700 susceptibility variants independently associated with PC risk (Zhang et al., 2020).

Most disease-associated risk loci are located in non-coding regions of the genome, raising the possibility that the variants might influence gene expression through effects on transcription initiation, splicing or mRNA stability (Gallagher and Chen-Plotkin, 2018). Germline genetic variants, such as single nucleotide polymorphisms (SNPs) in genes encoding for micro-RNAs (miRNAs), or in 3 prime untranslated regions (3'UTRs) of the corresponding binding sites can affect miRNA transcription and the mRNA-miRNA interaction, with the consequent alteration of gene expression (Landi et al., 2012; Iuliano et al., 2013; Ryan, 2017). miRNAs have been shown to participate in the development of PC, by modulating multiple cellular processes (Yonemori et al., 2017; Rawat et al., 2019). miR-217, miR-96, and miR-126 have been shown to regulate *KRAS*, which is the signature mutation gene in pancreatic carcinogenesis (Yu et al., 2010; Zhao et al., 2010; Jiao et al., 2012). Several studies have identified that miRNA-related SNPs could be associated with a range of diseases, including PC, lung cancer, colorectal cancer, gastric cancer, multiple myeloma, breast cancer, and attention-deficit/hyperactivity disorder (Kupcinskas et al., 2014; Ryan et al., 2015; Zheng et al., 2016; Macauda et al., 2017; Petkevicius et al., 2017; Mosallaei et al., 2019; Abdi et al., 2020; Bahreini et al., 2020). In this study, we aimed at identifying SNPs in the mature miRNA genes and target sites, involved in PC development.

## Materials and Methods

### Study Populations

We utilized a two-phase (discovery and replication) approach. For the discovery phase, the study populations used were obtained from the Pancreatic Cancer Cohort Consortium (PanScan I-III) and the Pancreatic Cancer Case Control Consortium (PanC4), which provide the largest publicly available pancreatic cancer GWAS data of European ancestry. The data were downloaded from the NCBI database of genotypes and phenotypes (dbGaP; study accession numbers phs000206.v5.p3 and phs000648.v1.p1; project reference #12644). Each participating study (within PanScan I-III and PanC4) obtained informed consent from study participants, and approval from the responsible institutional review board (IRB), as described in the original papers (Amundadottir et al., 2009; Petersen et al., 2010; Wolpin et al., 2014; Childs et al., 2015; Zhang et al., 2016; Klein et al., 2018). Genotyping for PanScan was performed using the Illumina HumanHap550, Human 610-Quad, and OmniExpress arrays, for PanScan I, II, and III, respectively. Genotyping for the PanC4 GWAS was performed using the Illumina HumanOmniExpressExome-8v1 array. We conducted standard quality control of the genotype data and performed imputation using the Michigan Imputation Server^1^ (Das et al., 2016) and the Haplotype Reference Consortium (HRC, V.r1.1) reference panel (McCarthy et al., 2016) for the datasets separately. SNPs with low imputation quality [INFO score *r*^2^ < 0.7, minor allele frequency (MAF) <0.005 or call rate <0.9] were excluded after imputation. We subsequently merged the four imputed datasets and obtained a pooled dataset containing 8,769 cases and 7,055 controls for further analysis of 7,543,430 SNPs.

Summary statistics of the European Study of Digestive Diseases and Genetics (PanGenEU) were used for replication. PanGenEU has been described in detail elsewhere (Gomez-Rubio et al., 2017; Molina-Montes et al., 2020). Briefly, it is a case–control study conducted in Spain, Italy, Sweden, Germany, United Kingdom, and Ireland, between 2009 and 2014. IRB approval and written informed consent were obtained from all participating centers and study participants, respectively. DNA samples were genotyped using the Infinium OncoArray-500 K, and genotype imputation was performed using IMPUTE2 with 1,000 Genomes-phase 3 as reference panel (López de Maturana et al., 2021).

Samples from the PANDoRA consortium, mostly from European populations, were selected for genotyping as a replication set as well. Cases diagnosed with PC [mostly pancreatic ductal adenocarcinomas (PDAC), and 137 other exocrine pancreatic cancers] were collected from the PANDoRA consortium, which has been described previously (Campa et al., 2013b). Controls were collected in the same geographical regions as the cases, mostly in the context of the PANDoRA consortium. We also included additional German controls from ESTHER, and British and Dutch controls from the European Prospective Investigation on Cancer (EPIC),^2^ which already have available GWAS data. ESTHER is a prospective cohort with 9,940 participants recruited during a general health check-up between July 2000 and December 2002 in the Saarland region of Germany. EPIC is an ongoing prospective cohort study that recruited healthy volunteers from the general population in 10 European countries (Riboli et al., 2002). EPIC samples were genotyped in the context of a GWAS using the Illumina Human 660 W-Quad BeadChip array. All subjects provided written informed consent and the ethical approval for the PANDoRA study protocol (including for controls from ESTHER and EPIC cohorts) was received from the Ethics Commission of the Medical Faculty of Heidelberg University.

The description of the study populations is shown in Table 1.

**Table 1:** Description of the study populations.

	PanScan + PanC4		PanGenEU		PANDoRA	
	Cases	Controls	Cases	Controls	Cases	Controls
Male, %	54.4	54.2	64	36	54.1	51.4
Median age, (25th–75th percentile)	65 (55–75)	65 (55–75)	66 (57–73)	65 (55–75)	66 (58–73)	60 (50–68)
*N*	8,769	7,055	1,317	700	3,976	3,506
Total	15,824		2,017		7,482	

### SNP Selection

**Figure 1:**
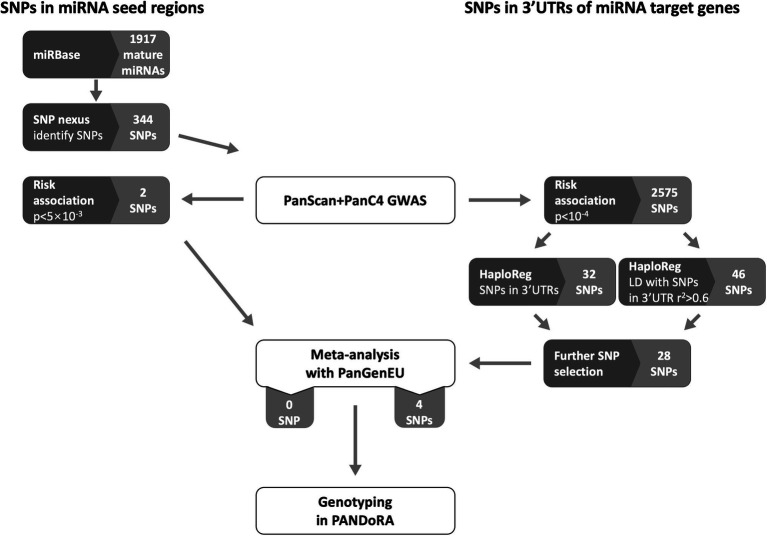
Flow chart of selection of SNPs located in the miRNA seed regions and the 3 prime untranslated regions (3'UTRs). Dark gray boxes indicate analytical processes (either database queries, association analysis, or SNP selection). Light gray boxes indicate numbers of miRNAs and SNPs in miRNA seed regions, or numbers of SNPs in 3'UTRs of miRNA target genes. White boxes indicate operations in data/sample sets.

We conducted a separate selection procedure for SNPs located in the miRNA seed regions (6–8 nucleotides at the 5' end of the mature miRNA) and SNPs located in the 3'UTRs of genes that are targeted by miRNAs. The flow chart of candidate SNP selection is shown in Figure 1.

The selection of SNPs located in mature miRNAs was processed using the database miRBase^3^ (Kozomara et al., 2019) and the web-tool SNPnexus (Dayem Ullah et al., 2013). We obtained a list of 1,917 mature miRNAs from miRBase (release 22.1). Then, SNPnexus^4^ was used to identify SNPs present in the seed region of those miRNAs by inserting the start and end position on the chromosome of each miRNA. The SNPs with MAF < 0.01 were excluded and 344 SNPs remained, among which 256 could be extracted and investigated in the PanScan and PanC4 populations. Around 10 SNPs were observed to be associated with pancreatic cancer risk with *p* < 0.05 (ranging from 2.74 × 10^−3^ to 0.049), and the top two most promising SNPs were forwarded to undergo a meta-analysis with the PanGenEU samples.

The selection of SNPs located in the 3'UTRs of miRNA target genes was conducted as follows: firstly, we analyzed the association of all 7.5 million SNPs in the PanScan and PanC4 pooled dataset with PC risk by logistic regression using an additive inheritance model adjusting for age, sex, and the top 10 principal components. Next, we extracted from the PanScan and PanC4 pooled dataset 2,575 SNPs that showed an association at *p* < 10^−4^. Among them, there were 32 SNPs predicted by HaploReg^5^ to be located in the 3'UTR regions, and 46 SNPs in linkage disequilibrium (LD, *r*^2^ > 0.6) with SNPs predicted by HaploReg to be located in the 3'UTR regions (Ward and Kellis, 2016). We performed further selection by removing SNPs located in known PC risk loci (*N* = 24 in six loci), SNPs that showed *p* ≥ 0.05 for association with PC risk in either PanScan or PanC4 (*N* = 18 in nine loci) and SNPs showing *p* > 5 × 10^−5^ (arbitrary threshold) for association with PC risk in the combined PanScan + PanC4 dataset (*N* = 8 in eight loci). Twenty-eight SNPs in 10 loci were further meta-analyzed with PanGenEU samples to select promising SNPs to be genotyped in PANDoRA samples.

### Genotyping

DNA of PANDoRA samples was isolated from whole blood using QIAamp DNA extraction kit (Qiagen) and distributed in 384-well plates for genotyping. For quality control, 8% of the samples were randomly replicated throughout the plates and no-template controls were used in each plate. Genotyping was performed using TaqMan (ABI, Applied Biosystems, Foster City, CA, United States) probes on the PCR system. Viia7 instrument and Viia7 software (Applied Biosystems, Foster City, CA, United States) were used to detect the genotypes. After calling all the genotypes, we performed several QC steps. Samples with more than one missing genotype were removed. Duplicated samples with more than one discordant genotype were excluded as well. Deviation from Hardy–Weinberg equilibrium (HWE) distribution was checked, in controls, in the overall population and by country. After QC, 3,976 cases and 3,506 controls collected from PANDoRA were included for further analysis, and all the genotyped SNPs were in HWE (*p* > 10^−3^).

### Statistical Analysis

To investigate the association of the genotyped SNPs in PANDoRA, we performed unconditional logistic regression adjusting for sex, age, and country of origin. We then performed meta-analyses using the fixed-effects model (or random-effects model when *p* < 0.05 in the heterogeneity test) between the discovery phase (PanScan and PanC4) and the replication phase (PanGenEU and PANDoRA). For the analysis with the genotyped SNPs in PANDoRA, age, sex, and genotypes had missing rates between 1 and 5%. Considering that missing data can have a significant effect on the results, we applied multiple imputation, which is a missing data method that provides valid statistical inferences under the missing at random (MAR) condition (Graham, 2009), with the R package “mice,” which imputes incomplete multivariate data by chained equations (van Buuren and Groothuis-Oudshoorn, 2011). Meta-analyses were performed after multiple imputations as well. Analyses were carried out with R V3.6.

### Bioinformatic Tools

We used the following tools/databases to explore the possible function of candidate SNPs: the Genotype-Tissue Expression (GTEx) project portal^6^ (accessed on 30 June 2020), to identify the possible effect of the SNPs on gene expression as expression quantitative trait loci (eQTL) or splicing quantitative trait loci (sQTL), HaploReg v4.1 (see Footnote 5), and RegulomeDB^7^ to test the regulatory potential (Boyle et al., 2012; Lonsdale et al., 2013; Ward and Kellis, 2016). The predicted effects of the SNPs (known loci and novel loci proposed in this study) on binding of miRNAs to their targets were obtained from the miRNASNP 3.0^8^ (Gong et al., 2015) and MirSNP^9^ (Liu et al., 2012) databases. We checked differential gene expression in cancer and normal tissue using the Gene Expression Profiling Interactive Analysis (GEPIA) database^10^ (Tang et al., 2017).

## Results

**Figure 2:**
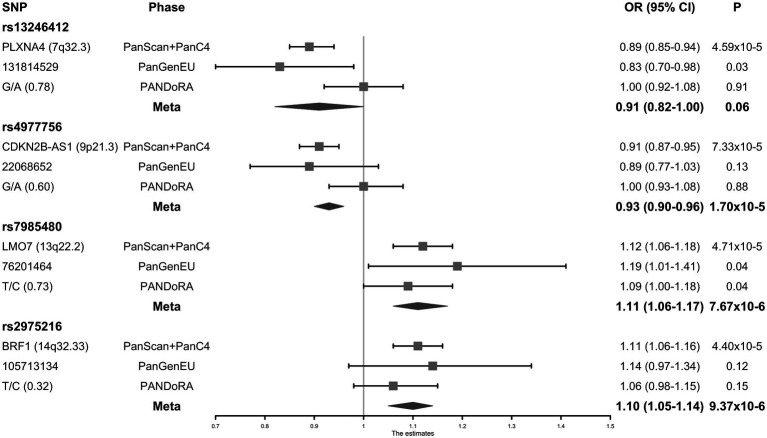
Forest plot of the associations between the four micro-RNA (miRNA)-related single nucleotide polymorphisms (SNPs) and the risk of pancreatic cancer (PC).

Meta-analysis for two SNPs in the miRNA seed regions and 28 SNPs selected by the miRNA target approach was performed with 1,317 PC cases and 700 controls from the PanGenEU study. The two miRNA seed SNPs (mir-182-rs76481776 and mir-4772-rs62154973) did not show a sufficiently significant association with PC risk (*p* = 5.02 × 10^−3^ and *p* = 4.64 × 10^−2^, respectively), and were not prioritized for replication in PANDoRA. On the contrary, four of the 28 SNPs selected by the miRNA target approach, which showed at least 5-fold lower value of *p* after adding PanGenEU were carried forward to the PANDoRA replication phase. The associations between these four SNPs and the risk of PC are shown in Figure 2 and Supplementary Table 1. rs7985480 was statistically significant (*p* < 0.05) in both PANDoRA and PanGenEU populations, and rs13246412 was observed to be associated with PC risk within the PanGenEU population. However, we did not observe genome-wide significant associations between the four SNPs and PC risk in the overall meta-analyses including all cases and controls. rs13246412 showed high heterogeneity in meta-analyses. The other three SNPs exhibited lower value of *p* in the meta-analysis in comparison with those observed in the discovery phase.

Eighty-seven putatively affected miRNAs were identified using the miRNASNP and mirSNP databases for four known PC risk loci (8q21.13, 13q12.1, 16q23.1, and 22q12.1) and four novel loci (Table 2). Each SNP has a different, independent effect on each different miRNA. Within the four genotyped SNPs, only rs13246412 is located in a 3'UTR. Thus, we predicted the miRNA binding effects for the other three genotyped SNPs using their linked SNPs located in the 3'UTR regions. rs1063192 (in LD with rs4977756, *r*^2^ = 0.78, D’ = 0.95) is predicted to have different effects across multiple miRNAs. Seven different miRNAs are predicted to bind *UCHL3* at the location of rs2274048 (in LD with rs7985480, *r*^2^ = 0.63, D’ = 1). When the allele changes from G to T, the miRNA-mRNA binding is predicted to lose strength for all of them. The T allele of rs4677, located in the 3'UTR of the gene *BTBD6*, is predicted to create a stronger binding site for miR-3622b-5p (rs2975216 is in strong LD with rs4677, *r*^2^ = 0.90, D’ = 1).

**Table 2:** Effect of SNPs on miRNA binding using miRNA target prediction databases.

Gene (Locus)	SNP in 3'UTR	Proxy (*r*^2^ for LD)^a^	miRNASNP		MirSNP			
			Gain	Loss	Create^b^	Enhance^b^	Decrease^b^	Break^b^
*PLXNA4* (7q32.3)	rs13246412^c^		miR-1207-5p, miR-1909-3p, miR-3128, miR-4720-5p, miR-4736, miR-4763-3p, miR-4799-3p, miR-5588-5p, miR-6132, miR-6722-3p, miR-6783-5p, miR-6798-5p, miR-6836-5p, miR-6868-5p, miR-7150 Allele-A	miR-10396a-5p, miR-10396b-5p, miR-1908-5p, miR-4525, miR-4706, miR-4723-5p, miR-4730, miR-4749-5p, miR-4787-5p, miR-5010–5p, miR-5698, miR-625-5p, miR-663a, miR-6770-3p, miR-6787-5p, miR-6870-5p, miR-7111-5p, miR-744-5p Allele-G	miR-1908, miR-4723-5p, miR-5698, miR-663a Allele-G	miR-4514, miR-940 Allele-G	miR-1207-5p, miR-3160-5p, miR-4514, miR-4763-3p, miR-940 Allele-A	miR-1909-3p Allele-A
*HNF4G* (8q21.13)^d^	rs2941484		miR-1277-5p, miR-16-2-3p, miR-195-3p, miR-5011-5p Allele-T	miR-16-1-3p, miR-222-5p, miR-5000-5p Allele-C	miR-1277-5p, miR-5011-5p Allele-T			miR-5000-5p Allele-C
*CDKN2B* (9p21.3)	rs1063192	rs4977756 (0.76)	miR-1297, miR-26a-5p, miR-26b-5p, miR-2681-3p, miR-382-5p, miR-4465, miR-4781-3p, miR-495-5p, miR-6516-3p, miR-96-3p Allele-A	miR-11399, miR-323b-5p, miR-361-5p, miR-494-5p, miR-410–5p Allele-G	miR-382-5p Allele-A		miR-889 Allele-G	miR-3074-3p, miR-323b-5p Allele-G
*PDX1* (13q12.2)^d^	rs9319402^c^		miR-4307, miR-5580-3p Allele-A					miR-4307, miR-5580-3p Allele-A
*UCHL3* (13q22.2)	rs2274048	rs7985480 (0.63)		miR-1297, miR-26a-5p, miR-26b-5p, miR-4465 Allele-G			miR-142-5p, miR-5590-3p, miR-561-3p Allele-G	
*BTBD6* (14q32.33)	rs4677	rs2975216 (0.88)			miR-3622b-5p Allele-T		miR-3665 Allele-C	
*CHST6* (16q23.1)^d^	rs9923834		miR-3690, miR-378j, miR-4254, miR-509-3p, miR-6839-5p Allele-A	miR-29b-1-5p, miR-29b-2-5p, miR-5087, miR-6740-5p Allele-T			miR-2392, miR-601 Allele-T	miR-1254, miR-3116 Allele-T
*CCDC117* (22q12.1)^d^	rs1222		miR-4754, miR-6850-5p Allele-C	miR-3161, miR-8075, miR-628-5p, miR-9718 Allele-A				

a SNPs not directly located in 3'UTRs of miRNA target genes, which were investigated for association with PC risk (see “Materials and Methods” section for more detail about SNP selection).

b Effects on miRNA binding site predicted by the MirSNP database: Create (creation of new miRNA recognition elements for miRNAs), Break (disrupt the miRNA binding sites completely), Increase (increase the binding affinity of the miRNA to existing binding sites), and Decrease (decrease the miRNA binding efficacy to existing binding sites).

c For these SNPs the miRNASNP and MirSNP databases provide opposite information about gain/loss of binding to some miRNAs.

d Known risk loci for pancreatic cancer, corresponding SNPs have not been genotyped in PANDoRA for replication.

Additionally, eQTL analysis suggests that the C allele of rs2975216 was associated with higher expression of *BTBD6* (*p* = 1.9 × 10^−7^) and of *BRF1* (*p* = 1.5 × 10^−5^) in pancreas. rs7985480-T was associated with increased expression of *LMO7-AS1* in adipose-subcutaneous tissue (*p* = 1.7 × 10^−14^). HaploReg and RegulomeDB did not show evidence for functional effect for these two variants. No significant eQTLs (*p* < 0.05) were seen for the SNPs at 7q32.3 (rs13246412) and 9p21.3 (rs4977756).

## Discussion

Several studies have shown the important role of miRNAs in various biological processes of pancreatic carcinogenesis, starting from the appearance of cancerous growth until its metastasis. The major differentially expressed miRNAs in PC tissue compared to adjacent non-malignant pancreatic tissue include upregulated miR-222, miR-21, miR-210, miR-221, miR-155, miR-196, miR-200a, miR-27a, and miR-212, and downregulated miR-200, miR-96, miR-217, miR-146, miR-245, miR-122, miR-31, miR-34, and miR-145 (Rawat et al., 2019). miRNA-related SNPs may affect miRNA function by influencing the miRNA biogenesis process or target interactions, causing alterations in gene expression that might be involved in the etiopathogenesis of PC.

In this study, we evaluated the associations between miRNA-related genetic variants (in miRNA genes or target binding sites) and PC risk. A possible limitation of the agnostic GWAS approach is that the typically applied genome-wide significance threshold (*p* < 5 × 10^−8^) might be overly conservative, which may lead to missing true disease risk loci. Our strategy in this study is to use functional annotation to prioritize SNPs, thereby using a low significance threshold for inclusion (*p* < 10^−4^), thus making it easier to potentially reach the genome-wide significance threshold with replication in additional samples. We surveyed the whole set of miRNA-related SNPs in the genome and selected four for full-scale analysis using a combined sample size of 14,062 pancreatic cancer cases and 11,261 controls. None of these SNPs reached genome-wide significance in the overall meta-analysis; however, we observed that the association between rs4977756 (9p21.3), rs7985480 (13q22.2), and rs2975216 (14q32.33) and PC risk was consistent in both the discovery and replication phase, with lower value of *p* in the overall meta-analyses compared with the discovery phase. In particular, rs7985480, in LD with rs2274048, located in the 3'UTR of *UCHL3*, was associated with pancreatic cancer risk with at least *p* < 0.05 in all datasets.

The binding efficacy of seven different miRNAs (miR-1297, miR-26a-5p, miR-26b-5p, miR-4465, miR-142-5p, miR-5590-3p, and miR-561-3p), which are predicted to bind *UCHL3* at rs2274048, is predicted to be weaker when the allele G is present, which would result in upregulation of *UCHL3*. This gene is involved in PC progression, and the highly expressed UCHL3-FOXM1 axis plays an important role in the oncogenesis and gemcitabine resistance (Song et al., 2019). The observed association that rs7985480-T (correlated with rs2274048-G) showed increased risk of PC is consistent with the predicted miRNA binding effects and the reported biological function. Additionally, GTEx shows that rs2274048 regulates *UCHL3* expression in several gastrointestinal tissues.

We cannot be completely sure that the underlying functional polymorphism for this observed association is rs2274048 or rs7985480, or even an unknown rare, untyped and not imputed variant. The analysis of sQTL in GTEx highlighted that the C allele of rs7985480 in the locus 13q22.2 regulates the alternative splicing of pre-mRNA of the gene *LMO7-AS1*, in pancreatic normal tissue (*p* = 1.4 × 10^−7^). These observations, while not conclusive, point to a possible regulatory function of this locus on chromosome 13.

SNP rs4977756 is intronic to *CDKN2B-AS1*, an RNA gene located within the *CDKN2B*-*CDKN2A* gene cluster at chromosome 9p21. Studies have reported that SNPs in the *CDKN2B* gene, which are in modest LD with rs4977756 (e.g., rs3217992, *r*^2^ = 0.41) are associated with the risk of PC and pancreatic neuroendocrine tumors (Campa et al., 2016a,b). The *CDKN2B-AS1* gene product is a functional RNA molecule that interacts with polycomb repressive complex-1 (PRC1) and polycomb repressive complex-2 (PRC2), leading to epigenetic silencing of other genes in this cluster. rs4977756 is in high LD with rs944801 (*r*^2^ = 0.84) that has been significantly associated with type 2 diabetes (T2D), a known risk factor for PC (Timpson et al., 2009; Zhao et al., 2017). All these evidences clearly support the involvement of this region in PC onset, probably through genetic variants regulating non-coding RNAs.

The most notable eQTL in this analysis was observed for rs2975216 at 14q32.33, where the risk-increasing C allele was associated with higher *BRF1* and *BTBD6* expressions in histologically unaffected pancreatic tissue samples. *BRF1* encodes one of the three subunits of the RNA polymerase III transcription factor complex. *BTBD6* is a protein coding gene, and it is involved in the innate immune system and class I MHC-mediated antigen processing and presentation pathways (as defined by GeneCards). rs297526 is not located in a 3'UTR region, but is in high LD with rs4677 (*r*^2^ = 0.90), located within the 3'UTR region of *BTBD6*. rs4677-T correlated with rs2975216-T, that in our data is associated with lower PC risk and is predicted to create a stronger binding site for miR-3622b-5p, which may repress the expression of *BTBD6*, consistent with the eQTL analysis of rs2975216. A recent transcriptome-wide association study for PC also identified *BTBD6* as a possible risk locus (Rawat et al., 2019; Zhong et al., 2020). Hence, we investigated the transcriptional data of *BTBD6* from the GEPIA database, and we found that the expression levels of *BTBD6* were indeed higher in PC tissues than in normal pancreas tissues. This suggests that increased expression of *BTBD6*, either by somatic events or constitutively through genetic polymorphisms, could be implicated in the etiology of PC.

In our study, four known risk loci are predicted to have different effects across multiple miRNAs. This shows that our approach is helpful to step from the statistical associations into the functional understanding of biological mechanisms underlying the risk of disease.

The two-phase approach contributes to decreasing the possibilities of spurious findings and offers a great strength. Although none of the SNPs we investigated with the full available sample size reached the genome-wide statistical significance, they remain attractive candidates. It may still be worth to include these relevant functional SNPs into polygenic risk scores for risk stratification.

## Data Availability Statement

The PanScan and PanC4 genotyping data are available from the database of Genotypes and Phenotypes (dbGaP, study accession numbers phs000206.v5.p3 and phs000648.v1.p1). The PANDoRA and PanGenEU primary data for this work will be made available to researchers who submit a reasonable request to the corresponding author, conditional to approval by the PANDoRA Steering Committee and Ethics Committees of the Medical Faculty of Heidelberg University, Germany, and of the Health Institute Carlos III (ISCIII), Madrid, Spain, respectively. Data will be stripped from all information allowing identification of study participants.

## Ethics Statement

The studies involving human participants were reviewed and approved by PANDoRA Steering Committee and Ethics Committee of the Medical Faculty of Heidelberg University (S-565/2015), Germany, and of the ISCIII, Spain. The patients/participants provided their written informed consent to participate in this study.

## Author Contributions

DC and FC conceived and designed the study. YL performed the lab work. YL, CC, MGe, and EL performed data quality control and statistical analyses. YL and CC drafted the manuscript. All other authors provided samples and data. All authors critically read, commented and approved the final manuscript.

## Conflict of Interest

The authors declare that the research was conducted in the absence of any commercial or financial relationships that could be construed as a potential conflict of interest.

## Publisher’s Note

All claims expressed in this article are solely those of the authors and do not necessarily represent those of their affiliated organizations, or those of the publisher, the editors and the reviewers. Any product that may be evaluated in this article, or claim that may be made by its manufacturer, is not guaranteed or endorsed by the publisher.

## Footnotes

^1^ https://imputationserver.sph.umich.edu

^2^ http://epic.iarc.fr/

^3^ http://www.mirbase.org

^4^ https://snp-nexus.org

^5^ https://pubs.broadinstitute.org/mammals/haploreg/haploreg.php

^6^ https://www.gtexportal.org

^7^ https://www.regulomedb.org/regulome-search/

^8^ http://bioinfo.life.hust.edu.cn/miRNASNP/

^9^ http://bioinfo.bjmu.edu.cn/mirsnp/search/

^10^ http://gepia.cancer-pku.cn
