# Supplementary material for: Association of Genetic Variants Affecting microRNAs and Pancreatic Cancer Risk
Source: Front Genet. 2021 Aug 30;12:693933. doi: 10.3389/fgene.2021.693933 (PMC8435735; doi:10.3389/fgene.2021.693933)
Supplement: Supplementary file 1 [file Table_1.DOCX]

Supplementary table 1. Associations of the four selected SNPs associated with miRNA binding effects with pancreatic cancer risk.

| SNP | Locus, Position, Gene^ψ^ | Major/Minor Alleles | EAF | Phase | OR (95% CI) | *P* |
| --- | --- | --- | --- | --- | --- | --- |
| rs13246412 | 7q32.3 | G/A | 0.78 | Discovery | 0.89 (0.85-0.94) | 4.59x10^-5^ |
|  | 131,814,529 |  |  | PanGenEU | 0.83 (0.70-0.98) | **0.03** |
|  | *PLXNA4* |  |  | PANDoRA | 1.00 (0.92-1.08) | 0.91 |
|  |  |  |  | Meta* | 0.91 (0.82-1.00) | 0.060 |
|  |  |  |  | Meta (MI)^#^* | 0.91 (0.83-1.00) | 0.060 |
| rs4977756 | 9p21.3 | G/A | 0.60 | Discovery | 0.91 (0.87-0.95) | 7.33x10^-5^ |
|  | 22,068,652 |  |  | PanGenEU | 0.89 (0.77-1.03) | 0.13 |
|  | *CDKN2B-AS1* |  |  | PANDoRA | 1.00 (0.93-1.08) | 0.88 |
|  |  |  |  | Meta | 0.93 (0.90-0.96) | 1.70x10^-5^ |
|  |  |  |  | Meta (MI)^#^ | 0.93 (0.90-0.96) | 9.33x10^-6^ |
| rs7985480 | 13q22.2 | T/C | 0.73 | Discovery | 1.12 (1.06-1.18) | 4.71x10^-5^ |
|  | 76,201,464 |  |  | PanGenEU | 1.19 (1.01-1.41) | **0.04** |
|  | *LMO7* |  |  | PANDoRA | 1.09 (1.00-1.18) | **0.04** |
|  |  |  |  | Meta | 1.11 (1.06-1.17) | 7.67x10^-6^ |
|  |  |  |  | Meta (MI)^#^ | 1.12 (1.07-1.17) | 3.03x10^-6^ |
| rs2975216 | 14q32.33 | T/C | 0.32 | Discovery | 1.11 (1.06-1.16) | 4.40x10^-5^ |
|  | 105,713,134 |  |  | PanGenEU | 1.14 (0.97-1.34) | 0.12 |
|  | *BRF1* |  |  | PANDoRA | 1.06 (0.98-1.15) | 0.15 |
|  |  |  |  | Meta | 1.10 (1.05-1.14) | 9.37x10^-6^ |
|  |  |  |  | Meta (MI)^#^ | 1.10 (1.05-1.14) | 9.84x10^-6^ |

EAF: effect allele frequency observed in the European subjects of the 1000 Genomes project; OR: odds ratio; CI: confidence interval

Bold type denotes p<0.05

^ψ^ Cytogenetic regions and SNP position according to NCBI Human Genome Build 37

* The meta-analysis for this SNP showed *P* < 0.05 in heterogeneity test

^#^ Meta-analysis results after multiple imputation
